# Large-scale integrated single-cell analysis reveals molecular subtypes and microenvironmental remodeling in cutaneous T cell lymphoma

**DOI:** 10.64898/2026.10.22.761138

**Published:** 2026-10-25

**Authors:** Ryan J Reis, Eric Mou, Vincent Liu, Patrick M Brunner, Nicholas Borcherding, Ali Jabbari

## Abstract

Cutaneous T cell lymphoma (CTCL) comprises a clinically and molecularly heterogeneous group of skin-homing non-Hodgkin lymphomas for which robust biomarkers remain limited. Prior single-cell RNA sequencing (scRNA-seq) studies have provided important insights into CTCL biology, but many of these prior studies have been limited by small sample sizes and focused on niche questions of interest. Additionally, many studies have suffered from pseudoreplication, resulting in inflated test statistics and reduced external validity. Here, we assembled and integrated scRNA-seq data spanning 244 unique samples and 132 patients across CTCL, healthy control (HC), and benign inflammatory dermatoses (BID) conditions, generating the most diverse atlas of CTCL to date. Following identification of malignant cell populations and heterogeneous, within-sample gene expression alterations, we applied pseudobulk aggregation and robust linear mixed-effects modeling to nominate recurrent changes across this highly heterogeneous patient cohort. Interrogation of the skin microenvironment identified significant compositional and cell type-specific expression changes in CTCL lesions as compared to control lesions and across clinical stages of disease. Finally, we used unbiased clustering to identify stable, molecularly defined subtypes of disease that were predictive of outcome in an independent dataset. Together, this study provides a rigorous analysis of the most diverse scRNA-seq dataset of CTCL ever compiled, nominating specific alterations in cell type composition and gene expression across malignant cell and microenvironmental populations with significant clinical implications.

